# Curcumin in Arthritis: Molecular Mechanisms, Preclinical Evidence, and Clinical Applications

**DOI:** 10.3390/ijms27114894

**Published:** 2026-05-28

**Authors:** Hechmi Toumi, Ahmad Almhdie-Imjabbar, Nada Ibrahim, Eric Lespessailles

**Affiliations:** 1 Translational Medicine Research Platform, PRIMMO, University Hospital Center of Orleans, 14 Avenue de l’Hôpital, 45100 Orleans, France; 2 Faculty of Sciences and Technology, University of Orleans, 1 Rue de Chartres, 45100 Orleans, France; 3 Department of Rheumatology, University Hospital Center of Orleans, 14 Avenue de l’Hôpital, 45100 Orleans, France

**Keywords:** curcumin, arthritis, osteoarthritis, rheumatoid arthritis, inflammation, bioavailability, nanomedicine, clinical trials

## Abstract

This review aims to provide a comprehensive and integrative evaluation of the therapeutic potential of curcumin in arthritis, focusing on its molecular mechanisms, preclinical evidence, and clinical applications. A systematic literature search was conducted across major databases, and a total of 165 studies were included in this review. Curcumin exerts multi-target effects on key pathogenic pathways involved in arthritis. At the molecular level, it inhibits inflammatory signaling pathways, particularly NF-κB, and reduces the production of pro-inflammatory mediators, including TNF-α, IL-1β, IL-6, COX-2, and PGE2. In parallel, curcumin modulates oxidative stress by enhancing antioxidant defenses, including superoxide dismutase (SOD) and glutathione (GSH), while reducing lipid peroxidation. It also regulates cell death pathways, including apoptosis, autophagy, and emerging mechanisms such as pyroptosis and ferroptosis, and preserves cartilage integrity by inhibiting matrix metalloproteinases and ADAMTS while promoting extracellular matrix components. Preclinical studies consistently demonstrate anti-inflammatory, antioxidant, and chondroprotective effects across in vitro and animal models. Clinical evidence, particularly in osteoarthritis, indicates improvements in pain and functional outcomes, with some studies suggesting efficacy comparable to nonsteroidal anti-inflammatory drugs. However, this evidence remains limited and should be interpreted with caution. However, variability in formulations and limited bioavailability remain key challenges influencing clinical outcomes. Overall, curcumin represents a promising multi-target therapeutic agent for arthritis. Further large-scale, well-designed randomized controlled trials using standardized and bioavailable formulations are required to confirm its efficacy and optimize its clinical application.

## 1. Introduction

Arthritis constitutes a major global health burden and remains one of the leading causes of chronic pain, disability, and reduced quality of life worldwide. The two most prevalent forms, osteoarthritis (OA) and rheumatoid arthritis (RA), differ in etiology but share common pathological features, including joint inflammation, cartilage degradation, and progressive functional impairment. OA is primarily a degenerative disease associated with aging and mechanical stress, whereas RA is an autoimmune disorder characterized by systemic inflammation and synovial hyperplasia [1,2]. In addition to OA and RA, other arthritic conditions such as gouty arthritis, ankylosing spondylitis, psoriatic arthritis, temporomandibular joint (TMJ) osteoarthritis, and hand osteoarthritis also contribute significantly to morbidity and healthcare burden.

Current pharmacological management of arthritis relies largely on nonsteroidal anti-inflammatory drugs (NSAIDs), corticosteroids, and disease-modifying antirheumatic drugs (DMARDs). Although these therapies can alleviate symptoms and, in some cases, slow disease progression, their long-term use is often associated with significant adverse effects, including gastrointestinal, cardiovascular, renal, and immunological complications [3]. These limitations have driven increasing interest in alternative or complementary therapeutic strategies that are both effective and safer for long-term use.

It should be noted that the concept of DMARDs has primarily been developed for inflammatory rheumatic diseases, such as rheumatoid arthritis. In contrast, osteoarthritis is not traditionally treated with DMARDs, and therapeutic strategies in OA are generally focused on symptom relief and, more recently, on the development of structure-modifying agents. Therefore, the potential role of curcumin as a disease-modifying agent should be interpreted differently depending on the specific type of arthritis.

Curcumin, a natural polyphenolic compound derived from the rhizome of turmeric (Curcuma longa), has attracted considerable attention due to its broad spectrum of biological activities. Extensive experimental and clinical studies have demonstrated that curcumin possesses anti-inflammatory, antioxidant, immunomodulatory, and chondroprotective properties relevant to arthritis management [4,5]. At the molecular level, curcumin modulates multiple signaling pathways implicated in arthritis pathogenesis, including nuclear factor-κB (NF-κB), pro-inflammatory cytokines such as tumor necrosis factor-α (TNF-α) and interleukin-1β (IL-1β), and matrix-degrading enzymes such as matrix metalloproteinases (MMPs) [1,6].

Clinical studies further support the therapeutic potential of curcumin in arthritis. Randomized controlled trials have reported significant improvements in pain and functional outcomes in patients with knee osteoarthritis treated with curcumin compared with placebo [7,8]. Moreover, curcumin has been shown to be comparable to conventional NSAIDs such as ibuprofen in reducing symptoms, with a potentially more favorable safety profile [3]. Additional studies have also demonstrated its efficacy in reducing oxidative stress and improving clinical symptoms in osteoarthritis patients [9], as well as its beneficial effects in rheumatoid arthritis [10].

Despite these promising findings, the clinical application of curcumin is significantly limited by its poor bioavailability. Native curcumin exhibits low aqueous solubility, rapid metabolism, and limited systemic absorption, resulting in insufficient plasma and tissue concentrations following oral administration [11]. To address these challenges, numerous strategies have been developed to enhance curcumin bioavailability, including co-administration with bioenhancers such as piperine [7], formulation into phospholipid complexes [12], and the development of nanoparticle-based and lipid-based delivery systems [13,14].

Consequently, curcumin has been investigated in multiple therapeutic formats, including the free compound, bioavailability-enhanced formulations, nanotechnology-based delivery systems, synthetic derivatives, and combination therapies with conventional drugs or other bioactive compounds. These approaches aim to improve pharmacokinetic properties, enhance therapeutic efficacy, and enable targeted delivery to inflamed joint tissues [15,16].

The present review aims to provide a comprehensive and integrative overview of the role of curcumin in arthritis. Specifically, it synthesizes current evidence regarding its molecular mechanisms, immunomodulatory effects, formulation strategies, and clinical efficacy across different arthritic conditions. By consolidating findings from in vitro studies, animal models, and human clinical trials, this review seeks to clarify the therapeutic potential of curcumin and identify key challenges and future directions for its clinical translation.

To better understand the biological effects of curcumin described in this review, it is essential to consider its chemical structure and physicochemical properties.

### Chemical Structure and Physicochemical Properties of Curcumin

Curcumin (diferuloylmethane) is a natural polyphenolic compound derived from the rhizome of Curcuma longa. It is the principal curcuminoid responsible for the biological activity of turmeric and has been extensively investigated for its anti-inflammatory, antioxidant, and therapeutic properties.

Structurally, curcumin consists of two aromatic phenolic rings connected by a seven-carbon linker containing α,β-unsaturated carbonyl groups. This unique structure confers both hydrophobic and electrophilic properties, enabling curcumin to interact with a wide range of molecular targets. The phenolic hydroxyl groups contribute to its antioxidant activity through free-radical scavenging, while the conjugated diketone moiety allows interactions with cellular proteins via Michael addition reactions.

Curcumin exists in keto–enol tautomeric forms, with the enol form predominating under physiological and organic conditions, contributing to its chemical stability and biological activity. Despite its promising pharmacological profile, curcumin exhibits poor aqueous solubility, chemical instability at neutral and alkaline pH, rapid metabolism, and limited systemic bioavailability following oral administration. These pharmacokinetic limitations significantly restrict its clinical application.

To overcome these challenges, various formulation strategies have been developed, including nanoparticle-based systems, liposomes, phospholipid complexes, and co-administration with bioavailability enhancers such as piperine. These approaches aim to improve solubility, stability, and systemic exposure, thereby enhancing therapeutic efficacy.

Curcumin is generally well-tolerated and has demonstrated a favorable safety profile in both preclinical and clinical studies, even at relatively high doses. However, variability in absorption and metabolism remains a major limitation for its consistent clinical use.

Overall, the chemical structure and physicochemical properties of curcumin underpin both its broad biological activity and its pharmacokinetic limitations, particularly in terms of bioavailability. These characteristics are essential for understanding the molecular mechanisms and therapeutic effects described in the following sections.

## 2. Methods

### 2.1. Study Design

This review was conducted following the principles of systematic reviews and reported in accordance with the Preferred Reporting Items for Systematic Reviews and Meta-Analyses (PRISMA) guidelines [17]. The objective was to systematically identify, evaluate, and synthesize available evidence on the therapeutic role of curcumin in arthritis, including mechanistic, preclinical, and clinical studies.

### 2.2. Literature Search Strategy

A comprehensive literature search was performed across multiple electronic databases, including PubMed/MEDLINE, Scopus, and Web of Science, to identify relevant studies published up to March 2026.

The search strategy combined keywords and Medical Subject Headings (MeSH) related to curcumin and arthritis. The following terms were used:

- “curcumin” OR “curcuminoids” OR “*Curcuma longa*”
- AND
- “arthritis” OR “osteoarthritis” OR “rheumatoid arthritis” OR “gout” OR “ankylosing spondylitis” OR “psoriatic arthritis” OR “joint disease”

Additional studies were identified through manual screening of the reference lists of relevant articles.

### 2.3. Eligibility Criteria

Studies were selected according to predefined inclusion and exclusion criteria.

Inclusion criteria

- Original research articles (in vitro, animal, or human studies)
- Studies investigating curcumin or curcuminoids in any form (free compound, enhanced formulations, derivatives, or combinations)
- Studies focused on arthritis-related conditions, including:
  (1) osteoarthritis (OA)
  (2) rheumatoid arthritis (RA)
  (3) gouty arthritis
  (4) ankylosing spondylitis
  (5) psoriatic arthritis
  (6) temporomandibular joint osteoarthritis
  (7) hand osteoarthritis
- Articles published in peer-reviewed journals
- Articles written in English

### 2.4. Exclusion Criteria

- Review articles, editorials, and conference abstracts without original data
- Studies not related to arthritis or joint inflammation
- Studies not involving curcumin or its derivatives
- Retracted articles (e.g., withdrawn publications were excluded from analysis)

### 2.5. Study Selection Process

All retrieved records were imported into a reference management system, and duplicates were removed. Titles and abstracts were independently screened to identify potentially eligible studies. Full-text articles were then assessed for eligibility based on the inclusion and exclusion criteria.

Disagreements during the selection process were resolved through discussion and consensus.

### 2.6. Data Extraction

Data were extracted systematically from each included study using a standardized approach. The following information was collected:

- Author(s) and year of publication
- Type of study (in vitro, animal, clinical trial)
- Arthritis model or patient population
- Type of curcumin intervention (free, formulation, nanoformulation, derivative, combination)
- Dosage and route of administration
- Duration of treatment
- Main outcomes (e.g., inflammation markers, cartilage degradation, pain scores)
- Key findings.

### 2.7. Data Synthesis and Classification

Given the heterogeneity of study designs, interventions, and outcome measures, a qualitative narrative synthesis was performed rather than a quantitative meta-analysis.

The included studies (*n* = 165) were categorized into major thematic domains:

(1) Molecular and cellular mechanisms
(2) Immunomodulatory effects
(3) Formulation and drug-delivery strategies
(4) Curcumin derivatives and analogs
(5) Combination therapies
(6) Regenerative and cell-based approaches
(7) Disease-specific evidence
(8) Clinical outcomes

This classification enabled structured analysis and comparison across different levels of evidence.

### 2.8. Risk of Bias and Limitations

Due to the inclusion of heterogeneous study types (in vitro, animal, and clinical studies), a formal quantitative risk-of-bias assessment was not uniformly applicable. However, particular attention was paid to:

- study design (randomized vs. non-randomized)
- sample size
- duration of intervention
- reproducibility of results
- formulation variability

Potential sources of bias, including small sample sizes and variability in curcumin formulations, were considered when interpreting the findings.

### 2.9. PRISMA Flow Diagram

**Figure 1:**
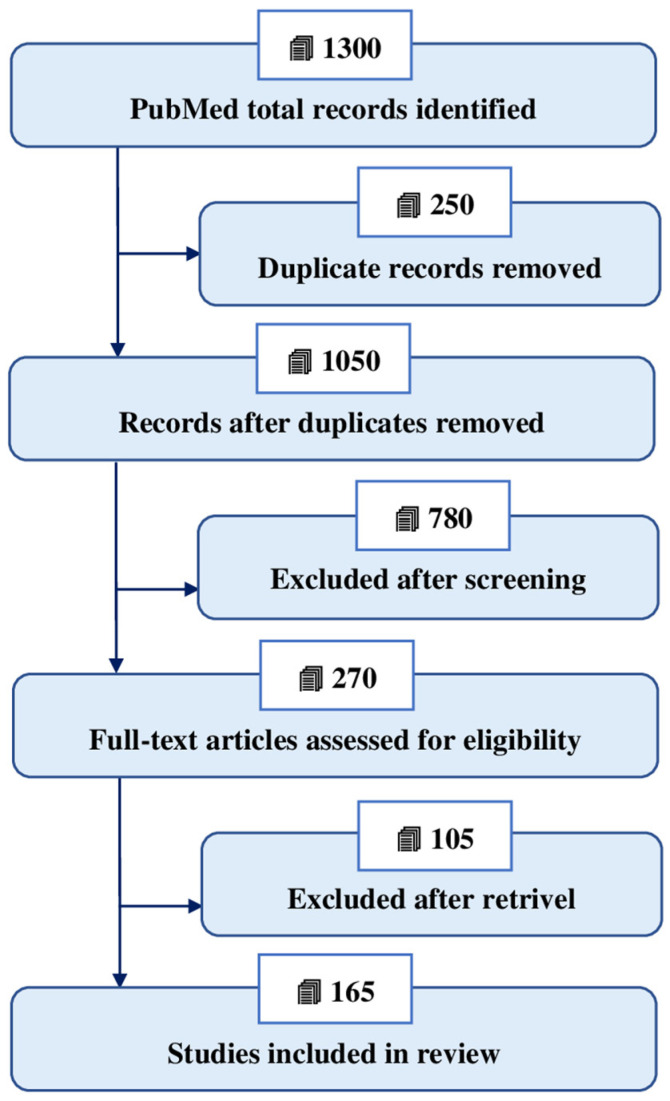
Flow diagram of the study selection process.

The study selection process is summarized using a PRISMA flow diagram (Figure 1), detailing the number of records identified, screened, excluded, and included in the final analysis [17].

Identification

- Records identified through database searching: *n* = 1248
- Additional records identified through other sources (reference lists, manual search): *n* = 52

Total records identified: *n* = 1300

Screening

- Records after duplicates removed: *n* = 1050
- Records screened (title/abstract): *n* = 1050
- Records excluded: *n* = 780

Eligibility

- Full-text articles assessed for eligibility: *n* = 270
- Full-text articles excluded: *n* = 105

Reasons for exclusion:

- Not related to arthritis: *n* = 28
- No curcumin intervention: *n* = 19
- Review/editorial/no original data: *n* = 34
- Insufficient data or unclear methodology: *n* = 16
- Retracted/withdrawn articles: *n* = 8

Included

- Studies included in qualitative synthesis: *n* = 165

## 3. Molecular and Cellular Mechanisms of Curcumin in Arthritis

Curcumin exerts pleiotropic effects on multiple molecular and cellular pathways involved in the pathogenesis of arthritis, including inflammation, oxidative stress, cell death regulation, and cartilage degradation. These effects are mediated through modulation of key signaling cascades, transcription factors, and effectors across joint tissues, including cartilage, synovium, and immune cells [1,5].

### 3.1. Anti-Inflammatory Signaling

Chronic inflammation is central to both Osteoarthritis and Rheumatoid Arthritis, with curcumin demonstrating broad inhibitory effects on pro-inflammatory signaling pathways.

A key mechanism involves suppression of the NF-κB pathway, a master regulator of inflammatory gene expression. Curcumin inhibits IκBα phosphorylation and degradation, thereby preventing NF-κB nuclear translocation and transcriptional activation of inflammatory mediators [1,2]. This leads to reduced expression of COX-2, MMPs, and cytokines such as TNF-α and IL-1β [5,6].

Curcumin also directly modulates cytokine networks. It suppresses IL-1β, TNF-α, and IL-6, which are critical drivers of synovial inflammation and cartilage degradation [5,18]. Clinical evidence supports these findings, with reductions in IL-6 and hs-CRP observed following curcuminoid supplementation in osteoarthritis patients [18]. Additionally, curcumin downregulates prostaglandin synthesis by inhibiting COX-2 and reducing PGE2 production [5].

Emerging evidence also indicates that curcumin influences IL-17-related pathways, particularly in autoimmune arthritis, although this remains less extensively characterized compared to TNF-α and IL-1β signaling [2].

Overall, curcumin acts as a multi-target anti-inflammatory agent, simultaneously modulating transcription factors, cytokines, and enzymatic mediators.

In addition to classical inflammatory pathways, recent studies have highlighted the NLRP3 inflammasome as a key regulator of inflammation in arthritis [19,20]. Activation of the NLRP3 complex leads to caspase-1 activation and the subsequent release of IL-1β and IL-18, contributing to synovial inflammation and cartilage damage. Emerging evidence suggests that curcumin can inhibit NLRP3 inflammasome activation through suppressing NF-κB signaling and reducing oxidative stress, thereby limiting inflammasome assembly and downstream inflammatory responses. These findings further support the role of curcumin as a multi-target anti-inflammatory agent.

### 3.2. Oxidative Stress Regulation

Oxidative stress contributes significantly to joint degeneration through reactive oxygen species (ROS)-mediated damage to cartilage and synovial tissues. Curcumin exhibits potent antioxidant activity by both direct scavenging of ROS and modulation of endogenous antioxidant systems.

In clinical settings, curcuminoid supplementation significantly increases antioxidant defenses, including superoxide dismutase (SOD) and glutathione (GSH), while reducing lipid peroxidation markers such as malondialdehyde (MDA) [9]. These findings demonstrate systemic attenuation of oxidative stress in osteoarthritis patients.

At the cellular level, curcumin inhibits ROS generation and oxidative damage in chondrocytes and inflammatory cells [4]. Mechanistically, curcumin also activates antioxidant pathways, such as NRF2/HO-1. However, this pathway is less frequently reported in arthritis-specific studies compared to its broader role in oxidative stress regulation.

By reducing oxidative stress, curcumin not only limits tissue damage but also indirectly suppresses redox-sensitive inflammatory pathways such as NF-κB.

Recent studies have also emphasized the role of SIRT1/AMPK signaling in mediating the antioxidant and anti-inflammatory effects of curcumin [21]. Activation of AMPK and its downstream target SIRT1 contributes to improved mitochondrial function, reduced oxidative stress, and inhibition of inflammatory signaling pathways. Curcumin has been shown to activate the AMPK/SIRT1 axis, thereby enhancing cellular resilience and reducing joint tissue damage in experimental models. This pathway represents an important link between metabolic regulation and inflammation in arthritis.

### 3.3. Regulation of Cell Death

Dysregulated cell death contributes to cartilage degeneration and synovial inflammation in arthritis. Curcumin modulates multiple forms of cell death, particularly apoptosis.

#### Apoptosis

Curcumin inhibits apoptosis in chondrocytes exposed to inflammatory stimuli by suppressing caspase-3 activation and restoring expression of structural proteins such as collagen type II [22]. At the same time, it can promote apoptosis in pathological cells such as hyperproliferative synoviocytes, thereby contributing to disease control [23].

Curcumin regulates multiple cell death pathways involved in arthritis pathogenesis, particularly apoptosis. In chondrocytes, curcumin inhibits mitochondrial apoptosis by modulating the balance between pro-apoptotic (Bax) and anti-apoptotic (Bcl-2) proteins, thereby reducing caspase-3 activation and preserving cell viability. This protective effect contributes to the maintenance of cartilage integrity under inflammatory conditions.

In contrast, curcumin may promote apoptosis in pathological synoviocytes, which are characterized by hyperproliferation and resistance to cell death in rheumatoid arthritis. This dual regulatory role highlights the context-dependent effects of curcumin in different joint cell populations.

Furthermore, the anti-apoptotic effects of curcumin are closely linked to its anti-inflammatory and antioxidant properties, as oxidative stress and inflammatory cytokines are key triggers of cell death in arthritis.

### 3.4. Emerging Cell Death Pathways: Pyroptosis and Ferroptosis

Curcumin has also been implicated in the regulation of emerging forms of regulated cell death, including pyroptosis and ferroptosis, which are increasingly recognized as important contributors to inflammatory joint diseases.

Pyroptosis is a highly inflammatory form of programmed cell death mediated by inflammasome activation, particularly the NLRP3 inflammasome. Activation of NLRP3 leads to caspase-1 activation, resulting in the maturation and release of pro-inflammatory cytokines such as IL-1β and IL-18. Although direct evidence of curcumin-mediated inhibition of pyroptosis in arthritis models remains limited, several studies suggest that curcumin suppresses NLRP3 inflammasome activation through inhibition of NF-κB signaling and reduction in reactive oxygen species (ROS). By attenuating these upstream signals, curcumin may reduce inflammasome assembly and subsequent inflammatory cell death, thereby limiting synovial inflammation and cartilage damage.

Ferroptosis, an iron-dependent form of cell death characterized by lipid peroxidation and glutathione depletion, has also been implicated in cartilage degeneration. Curcumin may exert protective effects against ferroptosis by enhancing antioxidant defenses and regulating key mediators such as glutathione peroxidase 4 (GPX4). By reducing ROS accumulation and lipid peroxidation, curcumin helps maintain cellular redox balance and prevent ferroptotic cell death in chondrocytes.

Although further studies are required to fully elucidate these mechanisms in arthritis-specific contexts, current evidence suggests that curcumin may indirectly regulate pyroptosis and ferroptosis through its combined anti-inflammatory and antioxidant effects.

Emerging forms of regulated cell death, including pyroptosis and ferroptosis, are increasingly recognized as potential contributors to inflammatory joint diseases. However, their roles in arthritis and their modulation by curcumin remain incompletely characterized.

### 3.5. Autophagy and Mitophagy

Autophagy is a critical cellular process involved in maintaining cellular homeostasis, particularly in chondrocytes, where it plays a protective role against stress-induced damage and cartilage degeneration. Dysregulation of autophagy has been implicated in the progression of osteoarthritis and other joint diseases.

Curcumin has been shown to modulate autophagic activity through multiple signaling pathways. Another mechanism involves inhibition of the PI3K/Akt/mTOR pathway, a key negative regulator of autophagy. By suppressing mTOR signaling, curcumin promotes autophagy initiation by activating upstream regulators such as ULK1 and Beclin-1, thereby enhancing cellular survival under inflammatory and oxidative stress conditions.

In chondrocytes, curcumin-induced autophagy contributes to the preservation of extracellular matrix components and reduces apoptosis. This effect is closely associated with decreased inflammatory signaling and improved cellular resilience. Curcumin has also been reported to influence transcription factors such as FoxO3, which regulate both autophagy and oxidative stress responses.

Mitophagy, the selective degradation of damaged mitochondria, is another important process for maintaining cellular function. Although direct evidence in arthritis remains limited, curcumin’s effects on mitochondrial function and oxidative stress suggest a potential role in regulating mitophagy through pathways such as PINK1/Parkin. By promoting mitochondrial quality control, curcumin may further protect chondrocytes from degeneration.

Overall, curcumin-mediated modulation of autophagy and mitophagy represents an important mechanism contributing to its chondroprotective and anti-inflammatory effects in arthritis.

In addition to classical signaling pathways, curcumin has been reported to exert epigenetic regulatory effects, including modulation of microRNAs, histone acetylation, and DNA methylation. These epigenetic mechanisms can influence the expression of genes involved in inflammation, oxidative stress, and cartilage metabolism. For example, curcumin-mediated regulation of specific microRNAs has been associated with reduced expression of pro-inflammatory cytokines and matrix-degrading enzymes. Although this field remains relatively emerging in arthritis research, epigenetic modulation represents an additional layer of curcumin’s pleiotropic activity.

### 3.6. Cartilage Catabolism and Extracellular Matrix Preservation

Cartilage degradation in arthritis is driven by increased activity of catabolic enzymes and loss of extracellular matrix components. Curcumin exerts strong chondroprotective effects by targeting these processes.

Curcumin inhibits the expression and activity of MMPs, including MMP-3 and MMP-9, which are responsible for collagen degradation [1,24]. It also suppresses ADAMTS enzymes, which degrade aggrecan, a key component of cartilage matrix [24].

At the same time, curcumin promotes the preservation of structural proteins, such as collagen type II and aggrecan, while enhancing the activity of chondrogenic transcription factors, such as Sox9 [2,22].

Additionally, curcumin inhibits hypoxia-related catabolic signaling pathways, including HIF-2α, which are implicated in osteoarthritis progression.

These combined effects result in reduced cartilage breakdown and improved structural integrity.

### 3.7. Synoviocyte and Macrophage Regulation

Synovial inflammation and immune cell activation are key contributors to arthritis progression. Curcumin modulates both synoviocytes and macrophages to reduce joint inflammation.

In fibroblast-like synoviocytes (FLS), curcumin inhibits proliferation, migration, and invasion by suppressing inflammatory signaling pathways and matrix-degrading enzymes [5]. It also reduces the production of pro-inflammatory mediators such as COX-2 and PGE2.

Curcumin further regulates immune responses by modulating macrophage activity. It reduces macrophage-driven inflammation by downregulating TNF-α and other cytokines, while also promoting a less inflammatory macrophage polarization [25].

Additionally, curcumin can inhibit macrophage proliferation and inflammatory signaling pathways, such as TNFR1, thereby reducing synovial inflammation [25].

Overall, curcumin acts on multiple interconnected pathways involved in arthritis pathogenesis. Its ability to simultaneously modulate inflammation, oxidative stress, cell survival, and cartilage metabolism supports its role as a multi-target therapeutic agent. These pleiotropic effects distinguish curcumin from conventional single-target therapies and underpin its growing interest in arthritis management.

## 4. Preclinical Evidence

Preclinical studies provide extensive evidence supporting the anti-arthritic effects of curcumin across in vitro systems and animal models. These studies demonstrate consistent anti-inflammatory, antioxidant, and chondroprotective properties and highlight the importance of formulation strategies to overcome curcumin’s limited bioavailability.

### 4.1. In Vitro Studies

In vitro models using chondrocytes, synovial fibroblasts, and immune cells have been instrumental in elucidating the molecular mechanisms of curcumin in arthritis.

Curcumin consistently suppresses inflammatory signaling in human articular chondrocytes, particularly by inhibiting IL-1β- and TNF-α-induced activation of NF-κB and downstream mediators such as COX-2 and MMPs [1,6]. This results in reduced production of inflammatory cytokines and preservation of extracellular matrix components.

In addition, curcumin protects chondrocytes from apoptosis and catabolic degradation. It restores collagen type II and β1-integrin expression while inhibiting caspase-3 activation under inflammatory conditions [22]. These findings highlight its direct chondroprotective role.

Studies on synovial fibroblasts (FLS) further demonstrate that curcumin reduces proliferation and inflammatory mediator production, including prostaglandins and cytokines [5]. Similarly, curcumin suppresses matrix-degrading enzymes, such as MMP-3 and inflammatory mediators, including nitric oxide and IL-6 [24].

Combination approaches have also shown synergistic effects. For example, curcumin combined with resveratrol enhances inhibition of NF-κB signaling and apoptosis in chondrocytes [6,26]. Likewise, combinations with collagen and green tea extract demonstrate enhanced suppression of inflammatory and catabolic mediators [24].

Furthermore, synthetic curcumin analogues exhibit enhanced potency. The diarylpentanoid derivative BDMC33 significantly inhibits NF-κB activation, MMP expression, and pro-inflammatory cytokines in synovial fibroblasts [27].

Overall, in vitro studies consistently show that curcumin modulates key cellular processes involved in arthritis, including inflammation, apoptosis, and matrix degradation.

### 4.2. Animal Models of Arthritis

Animal models provide critical evidence for the in vivo efficacy of curcumin in both inflammatory and degenerative arthritis.

In collagen-induced arthritis (CIA) and adjuvant-induced arthritis (AIA) models, curcumin significantly reduces joint swelling, inflammatory cytokine production, and histopathological damage [5,14]. These effects are associated with suppression of TNF-α, IL-1β, and NF-κB signaling.

Curcumin also demonstrates efficacy comparable to standard therapies in some models. For instance, intravenous curcumin showed therapeutic effects similar to those of methotrexate in rheumatoid arthritis models [14]. Additionally, curcumin reduces neutrophil activation and oxidative stress, further contributing to its anti-inflammatory effects [4].

Importantly, curcumin has been shown to improve clinical arthritis scores and reduce joint inflammation in animal models even with low systemic bioavailability, suggesting local or indirect mechanisms of action [28].

In osteoarthritis models, curcumin reduces cartilage degradation and inflammatory mediator expression, while preserving joint structure and function [15].

### 4.3. Role of Bioavailability and Drug Delivery Systems

A major limitation of curcumin is its poor oral bioavailability due to low solubility, rapid metabolism, and limited systemic absorption. Preclinical studies have therefore focused extensively on improving its delivery and efficacy.

#### 4.3.1. Nanoformulations

Nanotechnology-based delivery systems significantly enhance curcumin’s therapeutic effects. Lipid-core nanocapsules co-loaded with curcumin and resveratrol markedly improve anti-inflammatory efficacy and reduce joint edema compared to free compounds [13]. Similarly, nanoemulsion-based formulations increase skin penetration and improve anti-arthritic effects in topical applications [15].

#### 4.3.2. Phospholipid Complexes and Enhanced Formulations

Bioavailable formulations such as curcumin-phosphatidylcholine complexes demonstrate improved pharmacokinetics and enhanced biological activity [12]. These formulations increase systemic exposure and therapeutic potential.

#### 4.3.3. Chemical Derivatives

Structural modification of curcumin also improves its efficacy. Curcumin-diclofenac conjugates enhance bioavailability and exhibit superior anti-inflammatory activity compared to either compound alone [16]. Similarly, synthetic analogues show increased potency and stability [29].

#### 4.3.4. Adjuvants

Co-administration with bioavailability enhancers such as piperine significantly increases curcumin absorption and systemic effects, as demonstrated in both preclinical and clinical studies [9].

Although multiple strategies have been developed to improve the bioavailability of curcumin, their clinical relevance and level of supporting evidence vary considerably.

Among these approaches, formulations based on phospholipid complexes (e.g., curcumin–phosphatidylcholine) and bioavailability-enhanced preparations such as Theracurmin^®^ have demonstrated the most consistent clinical evidence, particularly in osteoarthritis, where they have been associated with improvements in pain and functional outcomes. These formulations benefit from improved absorption and more predictable pharmacokinetic profiles.

Co-administration with piperine has also been widely investigated and is known to significantly increase curcumin bioavailability by inhibiting hepatic and intestinal metabolism. However, concerns remain regarding potential drug interactions and variability in absorption, which may limit its widespread clinical applicability.

Nanoparticle-based and liposomal formulations represent promising advances, showing substantial improvements in bioavailability in preclinical studies. Nevertheless, clinical data for these approaches remain relatively limited, and issues related to cost, scalability, and regulatory approval may restrict their routine use.

Overall, while no single formulation can yet be considered definitively superior, current evidence suggests that standardized, bioavailability-enhanced formulations with clinical validation are the most practical and evidence-based options for therapeutic use. Future studies should directly compare these strategies to establish optimal formulations for different clinical settings.

### 4.4. Translational Relevance of Preclinical Findings

Preclinical evidence strongly supports curcumin as a multi-target agent capable of modulating key pathological processes in arthritis. The consistency of findings across in vitro and in vivo models reinforces its anti-inflammatory, antioxidant, and chondroprotective properties.

However, these studies also highlight important challenges. The discrepancy between strong preclinical efficacy and variable clinical outcomes is largely attributed to poor bioavailability and differences in formulation. This underscores the importance of optimized delivery systems for translating preclinical success into clinical benefit.

Collectively, preclinical studies demonstrate that curcumin effectively targets multiple aspects of arthritis pathogenesis, including inflammation, oxidative stress, and cartilage degradation, as summarized in Table 1. Advances in formulation strategies further enhance its therapeutic potential, paving the way for clinical application.

## 5. Clinical Evidence

Clinical studies evaluating curcumin in arthritis have primarily focused on Osteoarthritis (OA), with fewer trials in Rheumatoid Arthritis (RA). Overall, randomized controlled trials (RCTs) and observational studies suggest that curcumin may improve pain, function, and inflammatory markers, with a favorable safety profile (Table 2). However, heterogeneity in formulations, dosing, and study design complicates interpretation.

### 5.1. Randomized Controlled Trials in Osteoarthritis

Multiple RCTs have evaluated curcumin or curcuminoids in patients with knee osteoarthritis, generally demonstrating improvements in pain and functional outcomes.

A double-blind, placebo-controlled trial reported that curcuminoid supplementation (1500 mg/day for 6 weeks) significantly reduced pain (VAS), WOMAC scores, and functional impairment compared to placebo [7]. These findings support the clinical efficacy of curcumin in symptom relief.

Similarly, a study using a highly bioavailable formulation (Theracurmin^®^) showed significant reductions in knee pain and decreased reliance on NSAIDs over 8 weeks [8]. These results highlight the importance of formulation in achieving clinical benefit. While much focus remains on the knee, recent pilot RCT data suggest that low-dose curcumin (170 mg/day) may also provide significant pain relief and functional improvement in hand osteoarthritis over a 3-month period [34].

Curcumin has also been compared directly with standard pharmacological treatments. In a large multicenter trial, Curcuma domestica extract (1500 mg/day) demonstrated efficacy comparable to ibuprofen (1200 mg/day) in improving WOMAC scores, with fewer gastrointestinal adverse events [3]. This suggests that curcumin may serve as a safer alternative to NSAIDs for some patients.

Longer-term studies further support these findings. Supplementation with a curcumin-phosphatidylcholine complex (Meriva^®^) resulted in significant improvements in pain, mobility, and inflammatory markers over several months [12,31].

In addition to symptomatic improvement, curcumin may influence disease-related biomarkers. A clinical study demonstrated a reduction in the cartilage degradation marker Coll2-1 and a decrease in patient-reported disease activity following curcumin treatment [32].

### 5.2. Effects on Inflammation and Oxidative Stress in Patients

Clinical trials also provide evidence for curcumin’s biological effects on systemic inflammation and oxidative stress.

Curcuminoid supplementation has been shown to significantly reduce inflammatory markers such as IL-6 and hs-CRP, although effects on TNF-α and ESR are less consistent [18]. Interestingly, some studies report that clinical improvement does not always correlate directly with systemic inflammatory marker changes, suggesting additional mechanisms of action.

In parallel, curcumin improves antioxidant status in patients. A randomized controlled trial demonstrated increased levels of SOD and GSH, along with reduced MDA, indicating decreased oxidative stress [9]. These findings align with preclinical evidence of curcumin’s antioxidant properties.

### 5.3. Clinical Evidence in Rheumatoid Arthritis

Clinical evidence evaluating the effects of curcumin in rheumatoid arthritis (RA) remains limited but provides promising preliminary results. A randomized pilot study in patients with active RA demonstrated that curcumin supplementation (500 mg/day) significantly improved disease activity scores (DAS28), as well as tender and swollen joint counts. Notably, curcumin showed greater efficacy than diclofenac in this study and was well tolerated, with no reported adverse effects. These findings suggest that curcumin may exert clinically relevant anti-inflammatory effects in RA patients [10,35].

The therapeutic effects observed in RA are likely related to curcumin’s ability to modulate key inflammatory pathways involved in autoimmune responses, including inhibition of NF-κB signaling and suppression of pro-inflammatory cytokines such as TNF-α, IL-1β, and IL-6. In addition, curcumin may influence immune cell function, including T-cell activation and macrophage polarization, which are central to RA pathogenesis.

However, the current evidence base is limited by small sample sizes, short study durations, and heterogeneity in study design and curcumin formulations. Addressing these gaps, a 52-week Phase III RCT confirmed that chronic curcumin-piperine supplementation (2 g/day) is safe and well-tolerated [33]. Although it did not significantly outperform placebo in preventing flares during DMARD tapering, the study provides essential long-term safety data. Furthermore, variability in bioavailability may significantly affect clinical outcomes, making it difficult to establish standardized therapeutic protocols.

Consequently, while early clinical findings are encouraging, larger, well-designed randomized controlled trials using optimized and standardized formulations are required to confirm the efficacy of curcumin in RA and to better define its role in clinical practice.

### 5.4. Combination Therapies

#### Several Studies Have Explored Curcumin as Part of Combination Therapies

Co-administration of curcumin with NSAIDs such as diclofenac has shown potential additive or synergistic effects, although results are not always statistically significant [36]. These combinations may allow dose reduction of conventional drugs and minimize side effects.

In addition, curcumin combined with bioavailability enhancers such as piperine improves absorption and may enhance clinical outcomes [9]. Advanced delivery systems are emerging as a vital strategy. A recent study demonstrated that intra-articular nanoemulsified curcumin significantly reduces pro-inflammatory cytokines in osteoarthritis models [30].

### 5.5. Safety and Tolerability

Across clinical studies, curcumin demonstrates an excellent safety profile. Adverse events are generally mild and primarily gastrointestinal.

Compared with NSAIDs, curcumin is associated with fewer gastrointestinal side effects, particularly abdominal discomfort [3]. Long-term studies also report good tolerability without significant toxicity [12].

These findings support curcumin as a safe option for long-term management of chronic conditions such as osteoarthritis.

### 5.6. Limitations of Clinical Evidence

Despite promising results, several limitations should be considered.

First, there is substantial heterogeneity in curcumin formulations, including standard extracts, enhanced bioavailability products, and combination therapies, making direct comparison difficult. Second, many studies involve small sample sizes and short durations. Third, variability in outcome measures and endpoints limits consistency across trials.

Finally, the relationship between clinical outcomes and biological markers remains unclear, indicating that curcumin’s mechanisms in humans may extend beyond measurable systemic inflammation.

Clinical evidence supports the efficacy of curcumin in improving pain and function in osteoarthritis, with additional benefits on oxidative stress and inflammatory markers. Preliminary data in rheumatoid arthritis are encouraging but limited. While curcumin appears safe and potentially comparable to NSAIDs in some contexts, further large-scale, standardized trials are needed to confirm its clinical utility and optimize treatment strategies.

In addition to osteoarthritis and rheumatoid arthritis, other arthritic conditions such as gouty arthritis, ankylosing spondylitis, psoriatic arthritis, temporomandibular joint osteoarthritis, and hand osteoarthritis were included in the search strategy. However, the available evidence for these conditions remains limited and heterogeneous.

Most of the existing studies focus primarily on osteoarthritis and, to a lesser extent, rheumatoid arthritis, with relatively few well-designed experimental or clinical studies addressing these less common or more specialized conditions. As a result, the current review is largely centered on OA and RA, where the evidence base is more robust.

It is important to note that the majority of clinical studies evaluating curcumin in arthritis focus on knee osteoarthritis. This predominance likely reflects both the high prevalence of knee OA and its suitability as a model for clinical trials, where outcomes such as pain and function can be reliably assessed using standardized tools (e.g., VAS, WOMAC).

In contrast, clinical evidence for other joint sites (e.g., hand or temporomandibular joint osteoarthritis) and other arthritic conditions such as rheumatoid arthritis, psoriatic arthritis, or ankylosing spondylitis remains limited. This discrepancy appears to reflect a genuine gap in the literature rather than a limitation of the present search strategy, as these conditions were explicitly included in the inclusion criteria and search terms.

The relative scarcity of studies in these areas may be explained by greater disease heterogeneity, more complex pathophysiology, and challenges in clinical trial design and outcome standardization. Consequently, further research is needed to evaluate the efficacy of curcumin across a broader spectrum of arthritic conditions and joint sites.

Further research is needed to evaluate the potential role of curcumin in these additional arthritic conditions and to determine whether the mechanisms observed in OA and RA are applicable across different disease contexts.

## 6. Discussion

This review synthesizes evidence from 165 studies to evaluate the therapeutic potential of curcumin in arthritis. Overall, the findings indicate that curcumin exerts multifaceted biological effects targeting key pathological processes, including inflammation, oxidative stress, cartilage degradation, and immune dysregulation. These pleiotropic actions distinguish curcumin from conventional single-target therapies and support its role as a complementary or alternative strategy in arthritis management.

### 6.1. Integration of Mechanistic and Preclinical Evidence

One of the most consistent findings across studies is the ability of curcumin to modulate central inflammatory pathways, particularly NF-κB signaling, which regulates cytokines such as TNF-α, IL-1β, and IL-6 [1,5]. This upstream inhibition results in downstream suppression of COX-2, prostaglandins, and matrix-degrading enzymes, thereby reducing both inflammation and tissue destruction.

Preclinical studies strongly reinforce these mechanisms. In vitro experiments demonstrate that curcumin protects chondrocytes, inhibits synoviocyte activation, and suppresses catabolic enzymes such as MMPs and ADAMTS [1,24]. In vivo models further confirm reductions in joint swelling, inflammatory cytokines, and histological damage [5,14].

In addition, curcumin’s antioxidant effects—characterized by reduced ROS production and improved endogenous antioxidant defenses—contribute to its protective role in joint tissues [4,9]. These combined anti-inflammatory and antioxidant actions are particularly relevant given the interplay between oxidative stress and inflammation in arthritis pathogenesis.

Importantly, curcumin also influences cell survival pathways. It protects chondrocytes from apoptosis while promoting apoptosis in pathological synoviocytes, suggesting a context-dependent regulatory effect [22,23]. This dual action may help restore joint homeostasis.

### 6.2. Translation to Clinical Outcomes

Clinical studies largely corroborate preclinical findings, particularly in osteoarthritis. Multiple randomized controlled trials demonstrate improvements in pain, physical function, and quality of life following curcumin supplementation [7,8]. Notably, curcumin has shown comparable efficacy to NSAIDs such as ibuprofen, with fewer gastrointestinal adverse effects [3].

However, the translation from mechanistic effects to clinical outcomes is not always straightforward. While some trials report reductions in inflammatory biomarkers such as IL-6 and CRP, others show clinical improvement without significant changes in systemic inflammation [18]. This discrepancy suggests that curcumin may exert local joint-specific effects or act through pathways not fully captured by circulating biomarkers.

Evidence in rheumatoid arthritis remains limited but promising. A pilot study demonstrated superior improvement with curcumin compared to diclofenac [10], although larger trials are needed to validate these findings.

### 6.3. The Central Role of Bioavailability

A major challenge identified across studies is the poor bioavailability of curcumin, which limits its clinical efficacy. Native curcumin exhibits low solubility, rapid metabolism, and minimal systemic exposure.

To address this issue, numerous strategies have been developed, including nanoformulations, phospholipid complexes, and co-administration with absorption enhancers such as piperine [7,12,13]. These approaches significantly improve pharmacokinetics and enhance therapeutic outcomes in both preclinical and clinical settings.

Interestingly, some studies suggest that curcumin may exert biological effects despite low plasma levels, possibly through local intestinal or tissue-specific mechanisms [28]. This raises important questions about the relationship between systemic exposure and therapeutic activity.

### 6.4. Strengths and Limitations of the Evidence

**Figure 2:**
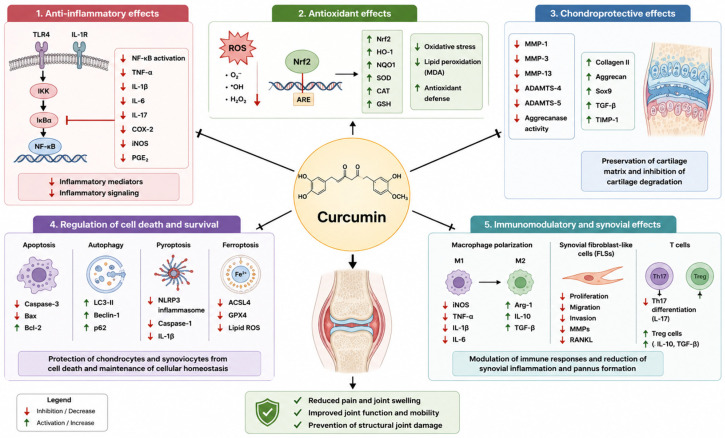
Proposed mechanisms underlying the protective effects of curcumin in arthritis. Curcumin exerts multi-target actions by inhibiting inflammatory signaling, reducing oxidative stress, protecting cartilage, regulating cell death and survival pathways, and modulating immune and synovial responses, ultimately leading to alleviation of arthritis symptoms and prevention of joint damage. Created using ChatGPT/OpenAI 5.4 for conceptual representation only.

A major strength of the current evidence is the consistency of mechanistic findings across different experimental models. The convergence of anti-inflammatory, antioxidant, and chondroprotective effects provides a strong biological rationale for curcumin use in arthritis (Figure 2).

However, several limitations must be acknowledged.

First, clinical studies show considerable heterogeneity in terms of formulations, dosing regimens, and outcome measures, making direct comparisons difficult. Second, many trials have relatively small sample sizes and short durations, limiting their statistical power and long-term relevance. Third, variability in bioavailability across formulations complicates the interpretation of efficacy.

Additionally, while preclinical models provide valuable insights, they do not fully replicate the complexity of human arthritis, particularly in chronic and multifactorial conditions such as Rheumatoid Arthritis.

### 6.5. Future Directions

Future research should focus on several key areas.

First, large-scale, well-designed randomized controlled trials are needed to confirm the efficacy of curcumin in different types of arthritis and to establish standardized dosing protocols. Second, greater emphasis should be placed on bioavailable formulations, as these appear critical for achieving consistent clinical benefits.

Third, mechanistic studies in humans are needed to better understand the relationship between curcumin’s molecular effects and clinical outcomes. This includes exploring emerging pathways such as autophagy, pyroptosis, and gut-joint interactions.

Finally, combination therapies involving curcumin and conventional drugs or other natural compounds may offer synergistic effects and improved therapeutic profiles.

In summary, curcumin represents a promising multi-target agent for arthritis, supported by robust preclinical evidence and encouraging clinical data. While challenges related to bioavailability and study heterogeneity remain, advances in formulation and a growing body of clinical research suggest that curcumin could play a meaningful role in the management of arthritis, particularly as a safe and complementary therapeutic option.

Despite its broad range of biological activities, curcumin is generally characterized by relatively modest potency compared to conventional pharmacological agents. Similar to other natural compounds such as resveratrol, its therapeutic effects are often limited by low bioavailability and the need for relatively high doses to achieve measurable clinical benefits.

Consequently, curcumin is unlikely to be used as a standalone therapy in the management of arthritis, particularly in moderate to severe disease. Instead, its most realistic clinical application may lie in its use as an adjunct or complementary therapy, aimed at enhancing treatment responses, reducing inflammation, and improving overall patient well-being.

This perspective is supported by clinical studies in which curcumin has been used alongside standard treatments, demonstrating favorable safety profiles and potential additive or supportive effects.

Therefore, while curcumin remains a promising multi-target agent, its role should be considered within an integrative therapeutic approach rather than as a primary pharmacological intervention.

In addition to its limited potency, the potential for drug–drug interactions should be carefully considered, particularly at higher doses of curcumin or when using bioavailability-enhanced formulations. Curcumin has been reported to modulate the activity of several cytochrome P450 enzymes, including CYP3A4, CYP1A2, and CYP2C9, as well as drug transporters such as P-glycoprotein. These effects may alter the pharmacokinetics of co-administered drugs, including commonly used medications in patients with arthritis, such as nonsteroidal anti-inflammatory drugs, corticosteroids, and disease-modifying antirheumatic drugs.

Furthermore, co-administration with bioavailability enhancers such as piperine, which inhibits hepatic and intestinal glucuronidation, may significantly increase systemic exposure to curcumin and potentially to other drugs, thereby increasing the risk of unintended interactions.

From a pharmacokinetic perspective, curcumin undergoes extensive first-pass metabolism, primarily through conjugation reactions such as glucuronidation and sulfation in the liver and intestine. It is rapidly converted into metabolites, including curcumin glucuronides and sulfates, which are generally considered less biologically active than the parent compound. In addition, reduction products such as tetrahydrocurcumin may contribute to its biological effects.

Overall, while curcumin is generally regarded as safe, its use at high doses or in combination with other therapies warrants careful consideration of potential drug–drug interactions. Further studies are needed to better characterize these interactions and their clinical relevance.

## 7. Conclusions

Curcumin has emerged as a promising multi-target therapeutic agent for arthritis, supported by a substantial body of preclinical and clinical evidence. Across experimental models, curcumin consistently demonstrates anti-inflammatory, antioxidant, and chondroprotective properties through modulation of key pathways such as NF-κB signaling, pro-inflammatory cytokines, and oxidative stress mediators [1,5].

Some clinical studies suggest that curcumin may provide improvements in pain and functional outcomes comparable to nonsteroidal anti-inflammatory drugs (NSAIDs). However, this observation is based on a limited number of trials and should be interpreted with caution. Further large-scale, well-designed randomized controlled studies are required to confirm these findings and to determine the extent to which curcumin may represent a viable alternative to NSAIDs in clinical practice.

Despite these promising outcomes, the clinical application of curcumin is constrained by its poor bioavailability, variability in formulations, and heterogeneity across studies. Advances in delivery systems, including nanoformulations and bioavailability enhancers, represent a critical step toward optimizing its therapeutic potential [12,13].

In conclusion, curcumin offers a safe and biologically plausible complementary approach for arthritis management. However, further large-scale, well-designed clinical trials using standardized and bioavailable formulations are essential to confirm its efficacy and establish its role in routine clinical practice.

## Figures and Tables

**Table 1:** Summary of representative preclinical studies evaluating the effects of curcumin in experimental models of arthritis.

Study	Model	Formulation	Dose/Route	Duration	Main Outcomes
Shakibaei 2005 [22]	Human chondrocytes	Free curcumin	In vitro	Short-term	↓ apoptosis, ↑ collagen II
Shakibaei 2007 [1]	Human chondrocytes	Free curcumin	In vitro	Short-term	↓ NF-κB, COX-2, MMP-9
Csaki 2009 [6]	Chondrocytes	Curcumin + resveratrol	In vitro	Short-term	Synergistic anti-inflammatory effect
Moon 2010 [5]	CIA mouse	Free curcumin	In vivo	21 days	↓ TNF-α, IL-1β
Comblain 2015 [24]	OA chondrocytes	Combination	In vitro	Short-term	↓ IL-6, MMPs
Zheng 2015 [14]	AIA rat	Nano-curcumin	Oral	14 days	↓ arthritis score
Coradini 2015 [13]	Rat model	Nano + resveratrol	In vivo	30 days	↓ edema
Naz 2015 [15]	OA model	Nanoemulsion	Topical	Variable	↑ cartilage protection
Jain 2014 [16]	Arthritis model	Curcumin-diclofenac	In vivo	Variable	↑ bioavailability
Hridayanka 2025 [30]	Animal model	Nano-curcumin	In vivo	Variable	↓ cytokines

↓ indicates decreased/downregulated/inhibited; ↑ indicates increased/upregulated/enhanced.

**Table 2:** Summary of representative clinical studies assessing the efficacy and safety of curcumin in patients with arthritis.

Study	Population	Design	Formulation	Dose mg/day	Duration	Main Outcomes
Belcaro 2010 [12,31]	OA	Clinical	Meriva	1000	8 months	↓ pain, ↑ mobility
Kuptniratsaikul 2014 [3]	Knee OA	RCT	Curcuma extract	1500	4 weeks	Comparable to ibuprofen
Panahi 2014 [7]	Knee OA	RCT	Curcuminoids	1500	6 weeks	↓ VAS, WOMAC
Nakagawa 2014 [8]	Knee OA	RCT	Theracurmin	NA	8 weeks	↓ pain
Henrotin 2014 [32]	OA	Clinical	Bioavailable	NA	Variable	↓ biomarker
Rahimnia 2015 [18]	Knee OA	RCT	Curcuminoids	1500	6 weeks	↓ IL-6
Panahi 2016 [9]	OA	RCT	Curcuminoids	NA	Variable	↑ antioxidants
Chandran 2012 [10]	RA	Pilot RCT	Curcumin	500	8 weeks	↓ DAS28
Bhat 2023 [33]	RA	RCT	Curcumin + piperine	Variable	52 weeks	Safe
Tuntiyatorn 2025 [34]	Hand OA	RCT	Curcumin	170	3 months	↓ pain

↓ indicates decreased/downregulated/inhibited; ↑ indicates increased/upregulated/enhanced.

## Data Availability

No new data were created or analyzed in this study. Data sharing is not applicable to this article.
